# Design, Synthesis and Cytotoxicity Evaluation of New 2-Aryl-5, 6-Dihydropyrrolo[2, 1-a]Isoquinoline Derivatives as Topoisomerase Inhibitors

**Published:** 2014

**Authors:** Samaneh Kakhki, Sorayya Shahosseini, Afshin Zarghi

**Affiliations:** Department of Medicinal Chemistry, School of Pharmacy, Shahid Beheshti University of Medical Sciences, Tehran, Iran.

**Keywords:** Pyrrolo[2, 1-a]isoquinolines, Topoisomerase Inhibitors, Molecular modeling, Cytotoxicity

## Abstract

Two set of 2-aryl-5, 6-dihydropyrrolo [2,1-a] isoquinolines were designed and synthesized to evaluate their biological activities as topoisomerase inhibitors. Cytotoxic activity of the synthesized compounds 4a-e and 7a-d was assessed against several human cancer cell lines, including MCF-7 (breast cancer cell), HepG2 (liver hepatocellular cells), A549 (adenocarcinomic human alveolar basal epithelial cells), T47D (Human ductal breast epithelial tumor cell line) and Hela (Human cervix cancer). According to our results, HepG2 seems to be the most sensitive cell line for these compounds with mean IC_50 _values ranging from 4.25 to 70.05 μM. Our results indicated that compound 7b exhibited the best potency against the tested cell lines. These results also suggest that pyrroloisoquinoline moiety constitutes a suitable scaffold to design new anti-proliferative agents.

## Introduction

**Figure 1:**
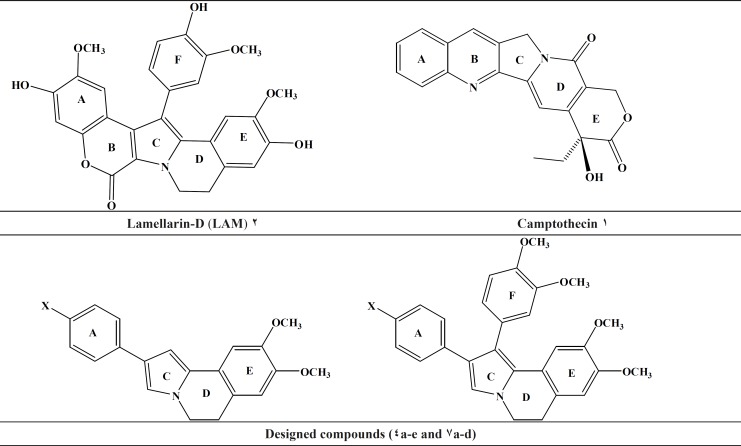
Topoisomerase inhibitors, lead compounds (Lamellarin-D), and our designed scaffold

**Figure 2:**
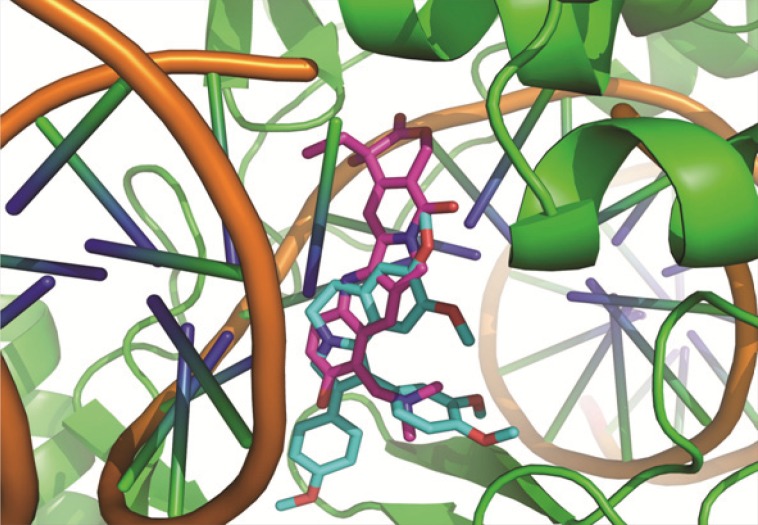
Molecular Modeling: good superimposition of the dihydroisoquinoline moiety of the synthesized compound 7b with the topotecan

According to the International Association for the Study of Pain, an estimated 6.6 million people from around the world die from cancer each year (1). Moreover, recent studies have also shown that cancer remains one of the world›s most serious health problems (2). Accordingly, there is an increased need to accelerate the progress of clinical cancer research, for the development of new anticancer agents. Topoisomerase I (Top I) is a crucial enzyme that regulate and adjust the topologic states of DNA and therefore is involved in all DNA processing steps, such as recombination, replication and transcription (3-5). As cancer cells tend to overexpress this enzyme, top it is a promising target for development of cytotoxic and chemotherapeutic drugs (6). Camptothecin (CPT) (Figure1), as a cytotoxic quinoline alkaloid is one of the earliest topoisomerase-targeting agents which was isolated from extracts of *Camptotheca acuminates *(7). It selectively inhibits Top I and exhibits strong antineoplastic acitivity. In 1985, Faulkner and co-workers isolated Lamellarin D (LAM-D) (Figure 1, 2) (8, 9), the polycyclic aromatic marine alkaloids, from the *Prosobranch Mollusc Lamellaria sp *which exhibit broad biological activities; including cell division inhibition, cytotoxicity, HIV-I integrase inhibition, and immuno modulatory activity (10-13). Although the lamellarins is potent and possess high cytotoxicity, it also suffers from well-identified drawbacks, including short duration of action, poor solubility and high toxicity (14-16). According to the existing SAR for LAM-D, its planar structure is thought to be one of the most important factors for topoisomerase inhibitory activity (17, 18). As part of ongoing program, the present study describes two set of new dihydropyrrolo [2, 1-a]isoquinolines possessing planar structure in conjunction with a substituent (H, OCH3, CH3, Cl, F) at the *para *position of phenyl ring (ring A) (Figure1, compounds 4a-e and 7a-d). Docking simulation was performed to position these compounds into topotecan binding site to determine the probable binding model, which may suggest the possible mechanism of the synthesized compounds. Cytotoxic activity of the synthesized compounds was also studied against MCF-7, Hep-G2, A549, T47D and Hela. 

## Experimental

*General*

All chemicals, reagents and solvents were purchased from Merck and Aldrich Chemical Company. Melting points were determined with a Thomas–Hoover capillary apparatus. 

Infrared spectra were acquired using a Perkin Elmer Model 1420 spectrometer. A Brucker FT-500 MHz instrument (Brucker Biosciences, Germany) was used to acquire 1HNMR spectra with TMS as internal standard. Chloroform-D and DMSO-D6 were used as solvents. Coupling constants (*J*) values are calculated in hertz (Hz) and spin multiplicities are given as s (singlet), d (double), t (triplet) and m (multi). The mass spectral measurements were performed on a 6410 Agilent triple quadrupole mass spectrometer (LCMS) with an electro spray ionization (ESI) interface. 

*Chemistry*

The DHIQ analogs (compound 4a-e and 7a-d) were synthesized as depicted in sheme1. 

Dihydroisoquinoline derivatives were prepared starting from β-arylethylamine in three steps. Treatment of β-arylethylamine with the acetyl chloride at 0 °C in anhydrous CHCl3 and 3,4-dimethoxy phenyl acetic acid under free solvent condition at 200 °C, provided desired amides 2 and 5 respectively(40%, 54%). The amides underwent a Bischler-Napieralski cyclization in the presence of POCl3, resulting in the formation of 3, 4-dihydroisoquinolines 3 and 6 (82%, 60%). The subsequent reaction of these dihydroisoquinolines with various phenacyl bromide derivatives in anhydrous ACN gave the residue was subjected to a plate chromatography to obtain the purified compounds 4a-e and 7a-d. All compounds were stable and kept in dry place at 25 °C. The structures of the synthesized compounds were confirmed by IR, 1HNMR and ESI-MS.

*3, 4-Dimethoxyphenethyl acetamide*

Yield: 40%; Yellow solid; Mp= 85 °C; IR (KBr): υ (cm-1) NH (3256), 1633 (C=O); LC-MS (ESI) *m/z *= 224 (M+1), 246 (M+23).

*6, 7-Dimethoxy-1-methyl-3, 4-dihydroisoquinoline*

Yield: 82%; Yellow solid; Mp= 166 °C; IR (KBr): υ (cm-1) 1603, 1517 (aromatic); LC-MS (ESI) *m/z *= 206 (M+1,100%).

*8, 9-Dimethoxy-2-phenyl-5, 6-dihydropyrrolo [2, 1-a]isoquinoline (4a)*

Yield: 55%; White solid; Mp= 134 °C; IR (KBr): υ (cm-1) 1606, 1556, 1502 (aromatic);

LC-MS (ESI) *m/z *= 306 (M+1, 100), 1HNMR (CDCl3/500MHZ): 3.05 (t, 2H, -CH2CH2N, *J *= 6.6 Hz), 3.93 (s, 3H, 8-OCH3), 3.98 (s, 3H, 9-OCH3), 4.10 (t, 2H, -CH2CH2N, *J*= 6.4 Hz), 6.68 (bs, 1H, pyrrole), 6.75 (s, 2H, 7-Ar-H and pyrrole), 7.11 (s, 1H, 10-Ar-H), 7.19 (t, 1H, Phenyl H4), 7.36 (t, 2H, Phenyl H3&H5), 7.58 (dd, 2H, Phenyl H2 and H6, *J*o = 8.2 Hz, *J*m = 1.2 Hz).

*8, 9-Dimethoxy-2-(4-methoxyphenyl)-5, 6-dihydropyrrolo [2, 1-a]isoquinoline (4b)*

Yield: 60%; White solid; Mp= 206 °C; IR (KBr): υ (cm-1) 1517, 1474 (aromatic); LC-MS (ESI) *m/z *= 336 (M+1, 100); 1HNMR (CDCl3/500MHZ): 3.04 (t, 2H, -CH2CH2N, J = 6.6 Hz), 3.71 (s, 3H, OCH3), 3.93 (s, 3H, 8-OCH3), 4.03 (s, 3H, 9-OCH3), 4.12 (t, 2H, -CH2CH2N, J= 6.4 Hz), 6.52 (bs, 1H, pyrrole), 6.70 (s, 1H, 7-Ar-H), 6.92 (d, 2H, 4-methoxyphenyl H3 and H5, *J *= 8.0 Hz), 7.08 (s, 1H, pyrrole), 7.29 (s, 1H, 10-Ar-H), 7.49 (d, 2H, 4-methoxyphenyl H2 and H6, *J *= 8.0 Hz).

*8, 9-Dimethoxy-2-p-tolyl-5, 6-dihydropyrrolo [2, 1-a]isoquinoline (4c)*

Yield: 52%; White solid; Mp= 152 °C, IR (KBr): υ (cm-1) 1602, 1507 (aromatic); LC-MS (ESI) m/z = 320 (M+1, 100); 1HNMR (CDCl3/500MHZ): 2.38 (s, 3H, CH3), 3.05 (t, 2H, -CH2CH2N, *J *= 6.6 Hz), 3.93 (s, 3H, 8-OCH3), 3.98 (s, 3H, 9-OCH3), 4.09 (t, 2H, -CH2CH2N, *J *= 6.6 HZ ), 6.68 (bs, 1H, pyrrole), 6.74 (s, 1H, 7-Ar-H), 7.11 (s, 1H, pyrrole), 7.18 (d, 2H, (4-methylphenyl H3 and H5, *J *= 7.9 Hz), 7.29 (s, 1H, 10-Ar-H), 7.47 (d, 2H, 4-methylphenyl H2 and H6, *J *= 7.9 Hz). 

*2-(4-Chlorophenyl)-8, 9-dimethoxy-5, 6-dihydropyrrolo [2, 1-a]isoquinoline (4d)*

Yield: 48%; White solid; Mp= 185 °C; IR (KBr): υ (cm-1) 1554, 1496 (aromatic); LC-MS (ESI) m/z = 340 (M+1, 100); 1HNMR (CDCl3/500MHZ): 3.05 (t, 2H, -CH2CH2N, *J*= 6.6 Hz), 3.93 (s, 3H, 8-OCH3), 3.98 (s, 3H, 9-OCH3), 4.10 (t, 2H, -CH2CH2N, *J*= 6.4 Hz ), 6.69 (s, 1H, pyrrole), 6.75 (s, 1H, 7-Ar-H)**, **6.96 (s, 1H, pyrrole), 7.09 (s, 1H, 10-Ar-H), 7.32 (d, 2H, 4-chlorophenyl H3 and H5, *J*= 8.4 Hz), 7.49 (d, 2H, (4-chlorophenyl H2 and H6, *J*= 8.4 Hz). 

*2-(4-Fluorophenyl)- 8,9-dimethoxy-5,6- dihydropyrrolo[2, 1-a]isoquinoline (4e)*

Yield: 55%; White solid; Mp= 186 °C; IR (KBr): υ (cm-1) 1598, 1513(aromatic); LC-MS (ESI) *m/z *= 324 (M+1, 100); 1HNMR (DMSO-d6/300MHZ): 2.99 (t, 2H, -CH2CH2N, *J*= 6.6 Hz), 3.87 (s, 3H, 8-OCH3), 3.92 (s, 3H, 9-OCH3), 4.04 (t, 2H, -CH2CH2N, *J*= 6.6 Hz), 6.58 (bs, 1H, pyrrole), 6.69 (s, 1H, pyrrole), 6.98 (m, 4H, (7-Ar-H, 10-Ar-H) and 4-fluorophenyl H3 and H5),7.44 (m, 2H, 4-fluorophenyl H2 and H6, *J *= 8.4 Hz).

*3,4-Dimethoxyphenethyl-2-(3,4-dimethoxyphenyl)acetamide (5)*

Yield: 54%; White solid; Mp= 120 °C, IR (KBr): υ (cm-1) NH (3320), 1631 (C=O); LC-MS (ESI) m/z = 359.9 (M+1), 381.8 (M+23).

*3,4-Dimethoxybenzyl-6,7-dimethoxy-3,4-dihydroisoquinoline (6)*

Yield: 60%, Yellow solid, Mp= 130 °C, IR (KBr): υ (cm-1)1597, 1559, 1506, LC-MS (ESI) m/z = 342 (M+1, 100%)

*1-(3, 4-Dimethoxyphenyl)-8, 9-dimethoxy-2-phenyl-5, 6-dihydropyrrolo [2, 1-a]isoquinoline (7a)*

Yield: 30%; White solid; Mp= 207 °C; IR (KBr): υ (cm-1) 1470, 1510 (aromatic); LC-MS (ESI) m/z = 442 (M+1, 100); 1HNMR (CDCl3/500MHZ): 3.09 (t, 2H, -CH2CH2N), 3.42 (s, 3H, OCH3), 3.73 (s, 3H, OCH3), 3.89 (s, 3H, 8-OCH3), 3.92 (s, 3H, 9-OCH3), 4.14 (t, 2H, -CH2CH2N), 6.69 (s, 1H, pyrrole), 6.72 (s, 1H, 7-Ar-H), 6.89-6.91 (m, 3H, 3,4-dimethoxy-phenyl H2 and H5 and H6), 7.18-7.22 (m, 5H, phenyl), 7.29 (s, 1H, 10-Ar-H).

*1-(3, 4-Dimethoxyphenyl)-8, 9-dimethoxy-2-(4-methoxyphenyl)-5, 6-dihydropyrrolo [2, 1-a] isoquinoline (7b)*

Yield: 48%; White solid; Mp= 188 °C; IR (KBr): υ (cm-1) 1550, 1503 (aromatic); LC-MS (ESI) m/z = 471 (M+1, 100); 1HNMR (CDCl3/500MHZ): 3.07 (t, 2H, -CH2CH2N, *J*= 6.5 HZ), 3.42 (s, 3H, OCH3), 3.74 (s, 3H, OCH3), 3.79 (s, 3H, OCH3), 3.89 (s, 3H, 8-OCH3), 3.92 (s, 3H, 9-OCH3), 4.11 (t, 2H, -CH2CH2N, *J*= 6.4 Hz ), 6.69 (s, 1H, pyrrole), 6.72 (s, 1H, 7-Ar-H), 6.77 (d, 2H, 4-methoxylphenyl H3&H5, *J *= 8.0 Hz), 6.90-6.95 (m, 3H, (3,4-dimethoxyphenyl H2 and H5 and H6), 7.12 (d, 2H, 4-methoxylphenyl H2 and H6, *J *= 8.0 Hz), 7.29 (s, 1H, 10-Ar-H).

*1-(3, 4-Dimethoxyphenyl)-8, 9-dimethoxy-2-p-tolyl-5, 6-dihydropyrrolo [2, 1-a]isoquinoline (7c)*

Yield: 47%; White solid; Mp= 192 °C, IR (KBr): υ (cm-1) 1511, 1490 (aromatic); LC-MS (ESI) *m/z *= 456 (M+1, 100); 1HNMR (CDCl3/500MHZ): 2.32 (s, 3H, CH3), 3.10 (t, 2H, -CH2CH2N), 3.41 (s, 3H, OCH3), 3.75 (s, 3H, OCH3), 3.89 (s, 3H, 8-OCH3), 3.92 (s, 3H, 9-OCH3), 4.13 (t, 2H, -CH2CH2N), 6.68 (s, 1H, pyrrole), 6.72 (s, 1H, 7-Ar-H), 6.90-6.91 (m, 3H, 3,4-dimethoxyphenyl), 7.03 (d, 2H, 4-methylphenyl H3 and H5, *J *= 8.1 Hz), 7.10 (d, 2H, 4-methylphenyl H2 and H6, *J *= 8.1 Hz), 7.29 (s, 1H, 10-Ar-H).

*2-(4-Chlorophenyl)-1-(3, 4-dimethoxyphenyl)-8, 9-dimethoxy--5, 6-dihydropyrrolo [2, 1-a] isoquinoline (7d)*

Yield: 53%; White solid; Mp= 210 °C, IR (KBr): υ (cm-1) 1507, 1485 (aromatic); LC-MS (ESI) m/z = 476 (M+1, 100); 1HNMR (CDCl3/500MHZ): 3.08 (t, 2H, -CH2CH2N, *J*= 6.4 HZ), 3.41 (s, 3H, OCH3), 3.76 (s, 3H, OCH3), 3.89 (s, 3H, 8-OCH3), 3.93 (s, 3H, 9-OCH3), 4.12 (t, 2H, -CH2CH2N, *J*= 6.4 Hz), 6.67 (s, 1H, pyrrole), 6.72 (s, 1H, 7-Ar-H), 6.88 (s, 2H, 10-Ar-H and 3,4-dimethoxyphenyl), 6.92 (s, 2H, 3,4-dimethoxyphenyl), 7.11 (d, 2H, 4-chlorophenyl H3 and H5, *J *= 8.3 Hz), 7.17(d, 2H, 4-chlorophenyl H2 and H6, *J *= 8.3 Hz).

**Scheme 1:**
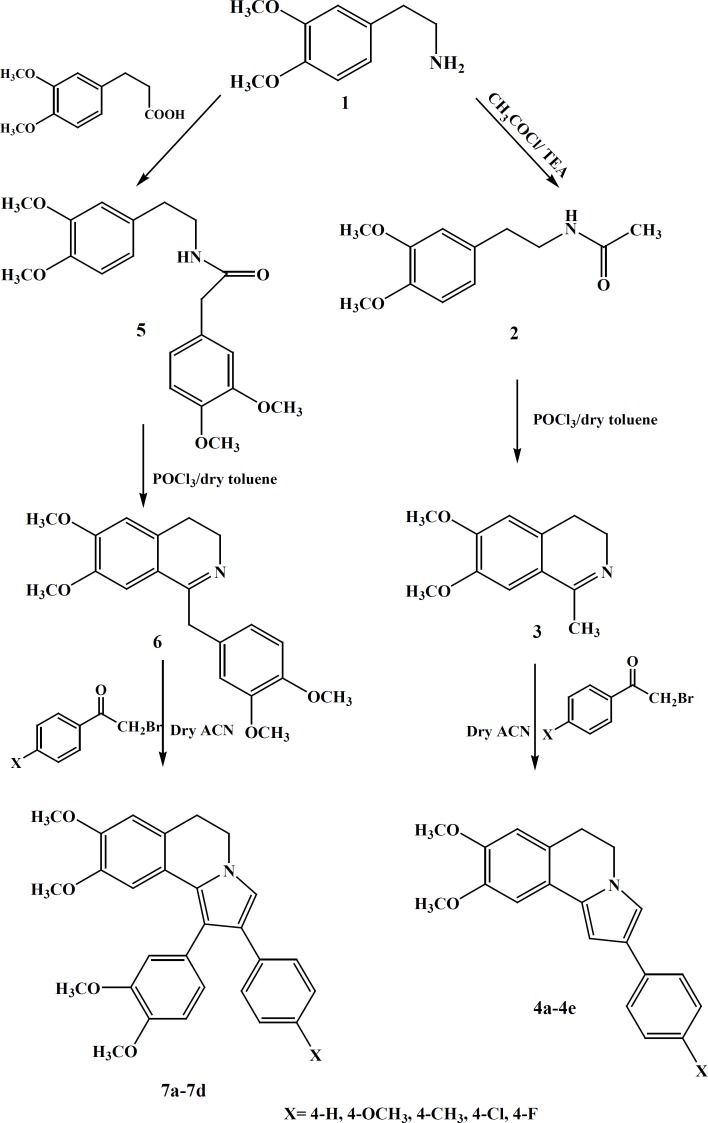
Synthesis of compounds 4a-4e and 7a-7d

*Cytotxicity*

Five human tumor cell lines were used to determine the cytotoxicity of the lamellarin derivatives**: **MCF7 (human breast cancer), Hep-G2 (human liver carcinoma), A549 (human lung cancer), Hela (human cervix cancer) and T47D (human breast cancer) cell lines were purchased from Pasteur Institute, Tehran, Iran. 

The cells were cultured in RPMI1640 medium at 37 °C under 5% CO2 supplemented with 10% fetal bovine serum (FBS), 100 U/mL penicillin and 100 μg/mL streptomycin. Cell viability was assayed by using a MTT method which is based on the reduction of 3-(4, 5-dimethylthiazol-2-yl)-2, 5-diphenyltetrazolium bromide (MTT) dye to purple formazan crystals by mitochondrial succinate dehydrogenase enzyme in living cells. The cells were seeded into 96-well plates at a concentration of 104 cells/well and allowed to incubate for 24 h. The cells were incubated with increasing concentrations of test compounds for 48h. At the end of each treatment period, 10 μL of MTT (5 mg/mL in PBS) was added to each well and the microplate was incubated at 37 °C for 4 h. The medium with MTT was removed and 100 μL DMSO was added to each well to dissolve the insoluble formazan crystals. Plates were incubated for 20 min at 37 °C and the optical densities were read at 570 nm with a reference wavelength of 630 nm as background using a spectrophotometer plate reader (Infinite® M200, TECAN). Doxorubicin was used as positive control and DMSO as the solvent of the test compounds. Data are presented as the mean of triplicate number of living cells and their capacity to reduce samples. IC50 was calculated by calibration curve using Prism software.

*Molecular modeling (docking) studies*

Docking studies were performed using Autodock Vina software for all the synthesized compounds to study their interactions with the topotecan binding site of topoisomerase were simulated. The X-ray crystal structure of the topoisomerase structure in complex with topotecan (entry code 1K4T) was obtained from the RCSB Protein Data Bank. All the compounds were built using hyperchem version 8 and subsequently minimized. The protein structure was prepared for docking using AUTODOCK Tool. Polar hydrogens were added and non-polar hydrogens were merged and finally Kollman united atom charge and atom type parameters were added to 1K4T. Grid map dimensions (28×28×28) were set surrounding active site. The energy minimized ligands were docked in binding site of topoisomerase. The quality of the docked structures was assessed by measuring the intermolecular energy of the ligand-enzyme assembly. 

## Results and Discussion

Two set of 2-aryl-5, 6-dihydropyrrolo [2, 1-a]isoquinoline were synthesized via multi-step reactions to prepare compounds 4a-e and 7a-d. All the compounds were confirmed using IR, ESI–MS and 1HNMR spectroscopies. Yields of the resulting compounds 4a-e and 7a-d ranged from 30% to 60%. 

Cytotoxic activity of the synthesized compounds 4a-e and 7a-d was assessed against several human cancer cell lines, including MCF-7 (breast cancer cells), HepG2 (liver hepatocellular cells), A549 (adenocarcinomic human alveolar basal epithelial cells), HeLa (human cervix cancer cells) and T47D (Human ductal breast epithelial tumor cell line). The results are shown in Table 1.

**Table 1:** *In-vitro *antiproliferative activity of compounds 4a-e and 7a-d based on MTT assay

**MTT assay 48 h IC**_50 _ **(μM)**					
**Compounds**	**MCF-7**	**A549**	**HeLa**	**HepG2**	**T47D**
4a	32.8 ± 0.04	38.4 ± 0.03	65.5 ± 0.06	9.5 ± 0.10	>100
4b	>100	44.6 ± 0.03	8.5 ± 0.04	31.2 ± 0.05	97.1. ± 0.08
4c	>100	9.2 ±0.05	29.3 ± 0.04	33.4 ± 0.04	>100
4d	37.3 ± 0.04	66.2 ± 0.03	18.8 ± 0.05	17.5 ± 0.03	>100
4e	>100	95.7 ± 0.04	49.9 ± 0.04	75.0 ± 0.05	>100
7a	81.1 ± 0.06	38.6 ± 0.02	40.1 ± 0.06	11.8 ± 0.03	97.6 ± 0.08
7b	9.4 ± 0.02	65.6 ± 0.04	22.5 ± 0.05	4.2 ± 0.04	>100
7c	>100	>100	69.9 ± 0.06	44.1 ± 0.03	>100
7d	>100	>100	54.9 ± 0.04	25.7 ± 0.05	>100
Doxorubicin	6.6 ± 0.02	0.06 ± 0.01	3.7 ± 0.31	1.1 ± 0.02	30.3 ± 0.04

IC50: drug concentration that inhibits cell growth by 50%.

According to our results HepG2 seems to be the most sensitive while T47D was the most resistant cell line to these compounds. All the compounds, possessed satisfactory activity against HepG2 with mean IC50 values raging from 4.25 to 75.02 μM. Our results indicated that all compounds exhibited weak to moderate cytotoxicity activity for HeLa (IC50; 8.5-69.9 μM) and A549 (IC50; 9.2-95.7 μM). However, compounds 7c and 7d did not show cytotoxicity activity for A549 cell line at 100 μM concentration. These results also indicated that the synthesized compounds did not have considerable anti-proliferative activity against MCF-7 and T47D cell lines except compound 7b which had good activity for MCF-7 (IC50; 9.4±0.02 μM). These results revealed that the compounds containing electron donating groups at *para *position of phenyl ring may improve the cytotoxicity activity. In addition, compounds containing an additional phenyl ring at position 2 of pyrrole ring (compound 7a-7d) did not show higher anti-proliferative activity than compound 4a-e which does not have this group. Based on these results, the presence of phenyl ring at position 2 of pyrrole ring is not essential for cytotoxicity activity. Also, our results indicated that compound 7b exhibited the best potency against the tested cell lines and the highest activity was observed against HepG2. Accordingly docking studies were performed for compound 7b in the active site of topoisomerase and compared with topotecan. As shown in Figure 2, compounds 7b showed the good superimposition with topotecan in the topoisomerase binding site. These data indicate that the mechanism of anti-proliferative activities of the designed molecules may be mediated through topoisomerase inhibition. These results also suggest that isoquinoline moiety constitutes a suitable scaffold to design new anti-proliferative agents. 
